# Increased expression of long-isoform thymic stromal lymphopoietin is associated with rheumatoid arthritis and fosters inflammatory responses

**DOI:** 10.3389/fimmu.2022.1079415

**Published:** 2023-01-16

**Authors:** Wanlin Li, Chenghui Liao, Jing Du, Jing Hu, Lu Wang, Xun Song, Zhendan He, Xiaohua Xiao, Liang Ye

**Affiliations:** ^1^Department of Pharmacy, International Cancer Center, Shenzhen University Medical School, Shenzhen, China; ^2^Department of Immunology, International Cancer Center, Shenzhen University Medical School, Shenzhen, China; ^3^Shenzhen Second People’s Hospital, The First Affiliated Hospital of Shenzhen University, Shenzhen, China; ^4^Department of Laboratory Medicine, Peking University Shenzhen Hospital, Shenzhen, China; ^5^Department of Respiratory Medicine, Shenzhen University General Hospital, Shenzhen University, Shenzhen, China; ^6^College of Pharmacy, Shenzhen Technology University, Shenzhen, China

**Keywords:** thymic stromal lymphopoietin, lTSLP, sTSLP, rheumatoid arthritis, inflammation

## Abstract

Thymic stromal lymphopoietin (TSLP) is a pleiotropic cytokine that is involved in the pathogenesis of inflammatory diseases and asthma, but the expression and biological implications of the existence of two isoforms, long TSLP (lTSLP) and short TSLP (sTSLP), in RA have yet to be elucidated. Here we report that lTSLP is the predominant TSLP isoform in RA and active RA, whereas sTSLP is the major TSLP isoform in inactive RA and healthy controls. lTSLP expression is associated with disease activity, including 28-joint Disease Activity Score (DAS28) and erythrocyte sedimentation rate (ESR), as well as proinflammatory cytokine expression, irrespective of other laboratory parameters. Importantly, lTSLP alone or combined with LPS promotes the expression of proinflammatory cytokines IL-1β, IL-6, and IL-8 in PBMCs of RA, but restrains anti-inflammatory cytokine IL-10 expression in PBMCs of RA. Furthermore, we found that STAT5 signaling is involved in lTSLP-induced inflammatory accumulation in PBMCs of RA. Therefore, these results highlight the clinical significance of lTSLP in RA pathology and inflammatory response in acute-phase disease, which may provide a therapeutic target for RA.

## Introduction

Rheumatoid arthritis (RA) is a chronic multisystem autoimmune disease that affects approximately 0.5-1.0% of adults and is characterized by chronic synovial inflammation and irreversible joint damage (1, 2). Although the etiology of RA is complex, with environmental, genetic, and immune factors all participating, the main RA pathological immune cause is thought to be dysregulated immune responses, with abnormally infiltrating inflammatory cells producing a variety of inflammatory cytokines (2, 3). Dysregulation of cytokine profiles has been proved and is frequently associated with an enhancement of proinflammatory cytokines (such as interleukin (IL)-1, IL-6, IL-8, and TNF-α) and attenuation of anti-inflammatory cytokines (such as IL-10 and TGF-β) in patients with RA (4). The abnormal production of these cytokines exacerbates the onset and duration of inflammation, ultimately resulting in irreversible joint destruction (5). Therefore, determining inflammatory cytokine signatures and suppressing pro-inflammatory cytokine expression could be promising strategies for RA in the prevention and treatment.

Thymic stromal lymphopoietin (TSLP) is a cytokine that belongs to the IL-7 family, which is predominantly produced by epithelial cells in mucosal tissues, also expressed by dendritic cells (DCs), monocytes, and mast cells (6–11). TSLP exerts pleiotropic effects by binding with a heterodimeric receptor complex composed of the TSLP receptor chain (TSLPR) and IL-7 receptor-α (IL-7Rα), which is widely expressed both on hematopoietic and non-hematopoietic cells (12–14). TSLP can drive allergic Th2 responses with the production of cytokines IL-4, IL-5, IL-13, and TNF-α *via* regulating OX40 ligand expression on DCs. Our work recently highlights the adjuvant activity of TSLP in inducing antiviral protective immunity (15, 16). Moreover, TSLP has been implicated in chronic inflammation, autoimmune diseases, and cancer (11, 17–21).

Recently, two distinct human isoforms of TSLP, a long isoform of TSLP (lTSLP) and a short isoform of TSLP (sTSLP), have been identified (22). The human lTSLP and murine TSLP share the same heterodimer receptor. sTSLP is composed of 63 amino acids and is homologous to the C-terminus of lTSLP (20, 23). Studies revealed that sTSLP is the primary isoform expressed under a steady-state condition and has anti-inflammatory and antimicrobial activities, whereas lTSLP is the major isoform expressed in an inflammatory state and promotes inflammatory responses (24–27). Because TSLP is a dual-functional cytokine with distinct isoforms, it is critical to determine its role in various diseases. However, the expression profiles and functions of two TSLP isoforms in RA patients remain unknown.

The present study aimed to assess lTSLP and sTSLP expression levels in RA patients and to investigate their correlation with RA activity, clinical and laboratory parameters, and inflammatory cytokines. We further sought to evaluate the regulatory role and mechanism of lTSLP in inducing the expression of inflammatory cytokine profiles in RA patients.

## Results

### lTSLP expression levels were upregulated in RA patients, particularly in active RA cohorts

**Figure 1:**
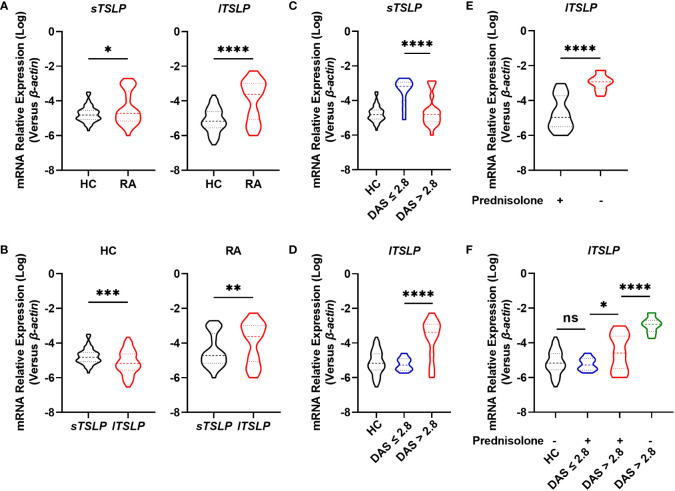
Comparison of mRNA levels of TSLP isoforms between RA patients and HC. **(A)** RT-qPCR analysis of the mRNA expression of sTSLP and lTSLP in PBMCs from RA patients (n = 68) versus HC (n = 66). **(B)** RT-qPCR comparison of sTSLP and lTSLP mRNA expression in PBMCs from HC and RA patients. **(C, D)** RT-qPCR analysis of sTSLP and lTSLP mRNA levels in active and inactive RA patients was compared to HC. **(E)** RT-qPCR analysis of the mRNA expression of lTSLP in RA patients with or without prednisolone treatment. **(F)** RT-qPCR analysis of lTSLP mRNA expression in HC and in active and inactive RA patients following prednisolone treatment. Data are depicted as a violin plot. **(A-F)** were analyzed by unpaired two-tailed Student’s t-test. *P < 0.05, **P < 0.01, ***P < 0.001, and ****P < 0.0001. ns, no significant.

To investigate whether TSLP isoforms (lTSLP and sTSLP) were involved in the pathogenesis of RA, we enrolled 68 RA patients and 66 age-and sex-matched healthy controls (HC), as detailed in **Table 1**. Initially, we revealed that both lTSLP and sTSLP mRNA expression levels were markedly increased in PBMCs of RA patients compared to healthy controls (**Figure 1A**). As it is well known that sTSLP is predominately expressed under steady-state conditions while lTSLP is preferentially expressed in inflammatory conditions, we assessed the expression difference of TSLP isoforms in the PBMCs of patients with RA and healthy controls. Consistent with these previous findings, we detected much higher mRNA expression levels of sTSLP than lTSLP in PMBCs of healthy controls, whereas lTSLP was the predominant isoform in RA cases (**Figure 1B**). These results appear to suggest that sTSLP is the major isoform of TSLP encountered under steady-state conditions, whereas lTSLP is more dependent on inflammatory environments. To verify this hypothesis, we classified RA patients into active groups (28-joint Disease Activity Score (DAS28) > 2.8) and inactive groups (DAS28 ≤ 2.8) according to RA disease activity. As shown in **Figure 1C**, sTSLP mRNA expression in PBMCs of active RA patients was notably lower than that in inactive RA cases. Conversely, RA patients in the acute activity phase had considerably higher lTSLP mRNA expression than inactive RA cases (**Figure 1D**). Afterward, RA patients were divided into prednisolone-treated and untreated groups to see whether effective RA treatment could influence lTSLP expression. We observed that prednisolone treatment greatly reduced lTSLP in RA patients (**Figure 1E**), particularly active RA patients (**Figure 1F**). These findings indicate that the upregulation of sTSLP in RA may serve as a feedback mechanism to maintain homeostasis and anti-inflammatory properties, whilst increased lTSLP levels may promote the inflammatory response in RA.

**Table 1:** Demographic and clinical characteristics of subjects.

Characteristics	RA patients	Healthy controls
NO. of cases	68	66
Female, n (%)	53 (77.94%)	45 (68.18%)
Male, n (%)	15 (22.06%)	21 (31.82%)
Age, years (range)	45.94 (22-73)	46.32 (24-70)
Disease duration (years)	8.5 ± 8.1	–
ESR (mm/h) (mean ± SD)	33.25 ± 27.41	–
RF concentration (IU/mL) (mean ± SD)	101.61 ± 110.99	–
CRP (mg/L) (mean ± SD)	5.37 ± 4.42	–
IgG (g/L) (mean ± SD)	12.16 ± 2.77	–
IgA (g/L) (mean ± SD)	2.08 ± 0.86	–
IgM (g/L) (mean ± SD)	1.461 ± 0.72	–
C3 (g/L) (mean ± SD)	0.98 ± 0.17	–
C4 (g/L) (mean ± SD)	0.21 ± 0.07	–
Anti-CCP (IU/mL) (mean ± SD)	271.79 ± 150.81	–
Anu A (IU/mL) (mean ± SD)	8.53 ± 8.77	–
Anti-dsDNA (IU/mL) (mean ± SD)	71.49 ± 56.86	–
G6PI (mg/mL) (mean ± SD)	0.20 ± 0.25	–
DAS28 (mean ± SD)	4.388 ± 1.549	–
Prednisolone responders, n (%)	38 (55.88%)	–
Prednisolone non-responder, n (%)	30 (44.12%)	–

Except where otherwise indicated, values are expressed as mean ± standard deviation. There were no significant differences between patients with RA and healthy controls in terms of age and sex.

ESR, erythrocyte sedimentation rate; RF, rheumatoid factor; CRP, C-reactive protein; IgG, IgA, IgM, immunoglobulin G, immunoglobulin A, immunoglobulin M; C3, complement component 3; C4, complement component 4; anti-CCP, anti-cyclic citrullinated peptide antibodies; Anu A, Anti-nucleosome antibody; Anti-dsDNA, anti-double stranded DNA antibody; G6PI, glucose 6 phosphate isomerase; DAS28, 28-joint Disease Activity Score.

### lTSLP expression was positively associated with DAS28 and ESR in RA patients

**Figure 2:**
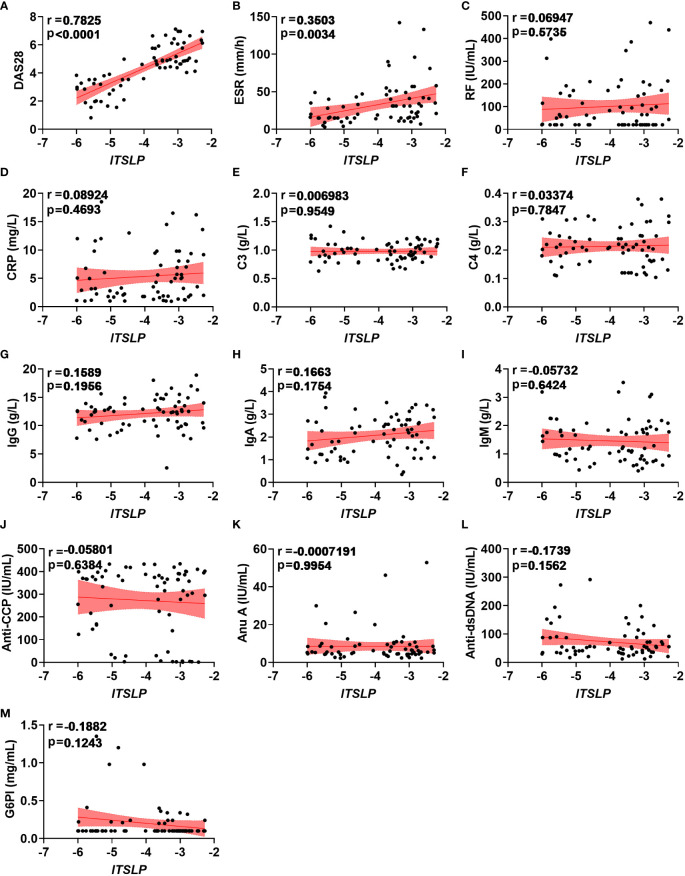
Correlations between lTSLP expression and RA clinical values. **(A-M)** The relationship analysis of *lTSLP* mRNA expression with laboratory values, including DAS28 **(A)**, ESR **(B)**, RF **(C)**, CRP **(D)**, C3 **(E)**, C4 **(F)**, IgG **(G)**, IgA **(H)**, IgM **(I)**, Anti-CCP **(J)**, Anu A **(K)**, Anti-dsDNA **(L)**, and G6PI **(M)**. The correlation was determined using Spearman’s non-parametric test. Each symbol represents an individual RA patient.

Since lTSLP mRNA expression, but not sTSLP mRNA expression, strongly coincided with disease activity progression, we wondered if lTSLP expression was linked with clinical and pathological parameters of RA. Interestingly, lTSLP mRNA levels were considerably and positively correlated with the DAS28 score (r = 0.7825, *P* < 0.0001) and the inflammatory marker erythrocyte sedimentation rate (ESR, r = 0.3503, *P* < 0.0034) (**Figures 2A, B**). However, there was no significant linear correlation between lTSLP mRNA levels and other laboratory values such as rheumatoid factor (RF, **Figure 2C**), C-reactive protein (CRP, **Figure 2D**), complement component 3 (C3, **Figure 2E**), complement component 4 (C4, **Figure 2F**), immunoglobulin G (IgG, **Figure 2G**), immunoglobulin A (IgA, **Figure 2H**), immunoglobulin M (IgM, **Figure 2I**), anti-cyclic citrullinated peptide antibodies (Anti-CCP, **Figure 2J**), Anti-nucleosome antibody (Anu A, **Figure 2K**), anti-double stranded DNA antibody (Anti-dsDNA, **Figure 2L**), and glucose 6 phosphate isomerase (G6PI, **Figure 2M**). Taken together, these results demonstrate that lTSLP expression is stridently associated with increasing RA severity and has the potential to be used as a RA diagnostic factor.

### lTSLP expression was tightly associated with inflammatory cytokines in RA patients

**Figure 3:**
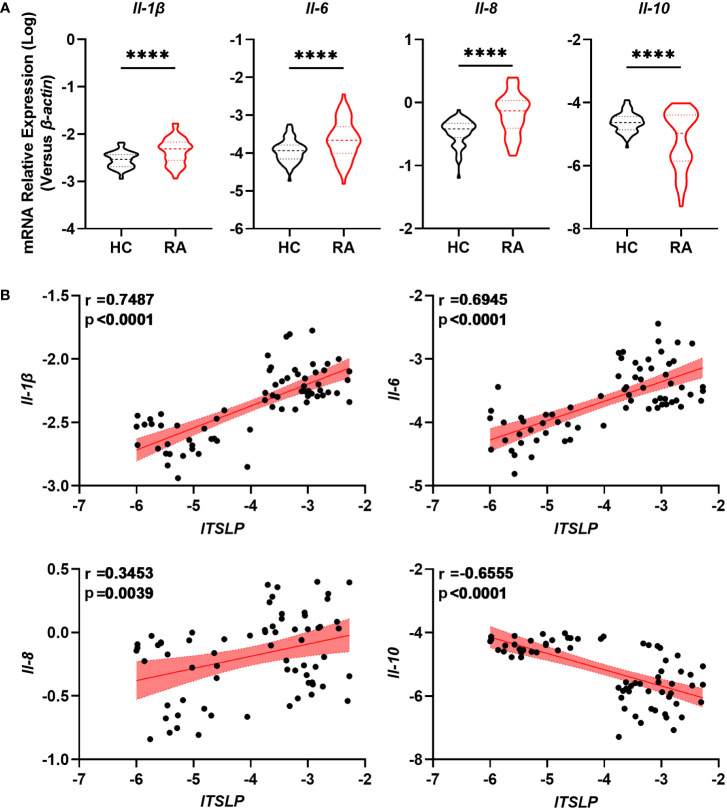
lTSLP expression in RA PBMCs correlates with pro- and anti-inflammatory cytokines. **(A)** RT-qPCR was used to detect the mRNA expression of pro-inflammatory cytokines IL-1β, IL-6, and IL-8, as well as the anti-inflammatory factor IL-10, in PBMCs from RA patients (n = 68) and HC (n = 66). The data was depicted as a violin plot. Statistical significance was determined by unpaired two-tailed Student’s t-test. ****P < 0.0001. **(B)** Correlations between lTSLP mRNA expression and the cytokines IL-1β, IL-6, IL-8, and IL-10 in PBMCs from RA patients. The correlations were evaluated using Spearman’s non-parametric test. Each symbol indicates a distinct patient.

Given that a disrupted balance of pro- and anti-inflammatory cytokines determines the progression and severity of RA, we researched the expression of cytokines IL-1β, IL-6, IL-8, and IL-10, as well as their association with lTSLP. We found that the pro-inflammatory cytokines including Il-1β, Il-6, and Il-8 mRNA levels in PBMCs of patients with RA were significantly higher than those of healthy controls (**Figure 3A**). By contrast, patients with RA showed decreased mRNA expression of the anti-inflammatory cytokine IL-10 compared to healthy controls (**Figure 3A**). Importantly, lTSLP mRNA levels in patients with RA were positively correlated with IL-1β mRNA levels (r = 0.7487, *P* < 0.0001), IL-6 mRNA levels (r = 0.6945, *P* < 0.0001), and IL-8 mRNA levels (r = 0.3453, *P* = 0.0039), respectively (**Figure 3B**). In contrast, lTSLP mRNA levels were negatively correlated with IL-10 mRNA levels in RA patients (r = -0.6555, *P* < 0.0001) (**Figure 3B**). As a result, these results suggest that lTSLP expression in RA patients is closely related to the pro- and anti-inflammatory milieu.

### lTSLP promotes inflammatory cytokine expression in RA involving STAT5 activation

**Figure 4:**
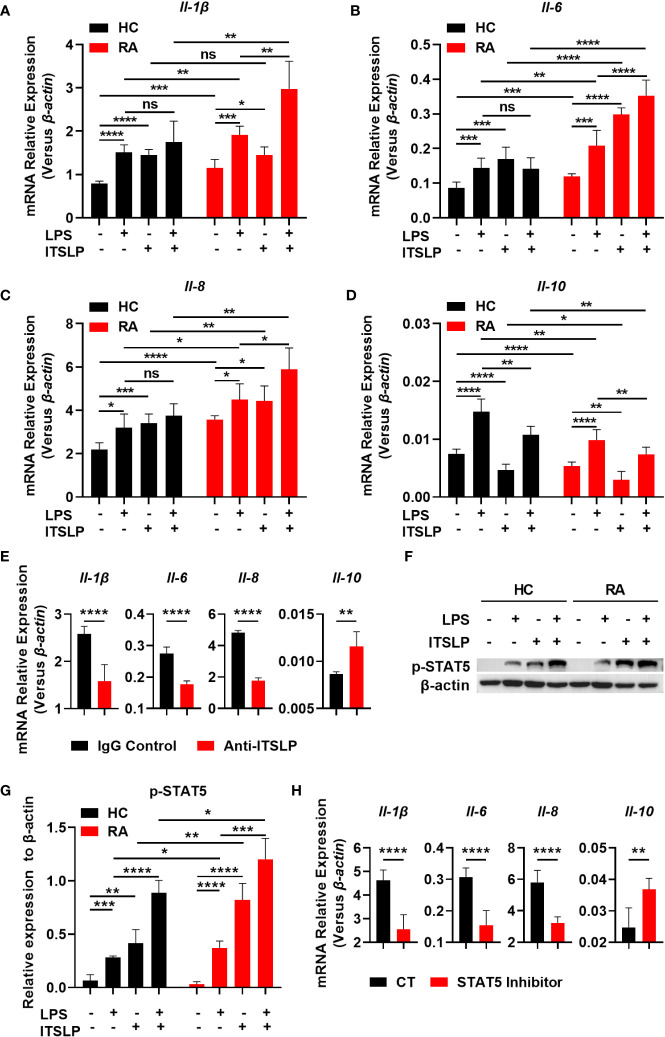
lTSLP induces pro-inflammatory cytokine expression in PBMCs by triggering STAT5 activation. **(A-D)** PBMCs from RA patients and healthy controls (HC) were cultured for 24h with or without LPS (100 ng/mL) or lTSLP (100 ng/mL). Relative mRNA levels of *Il-1β* **(A)**, *Il-6* **(B)**, *Il-8* **(C)** and *Il-10* **(D)** were analyzed by RT-qPCR. **(E)** PBMCs from RA patients were treated 24h with 0.25 µg/ml anti-TSLP neutralizing antibody or an IgG antibody control, and the relative mRNA levels of *Il-1β*, *Il-6*, *Il-8*, and *Il-10* were analyzed by RT-qPCR. **(F)** PBMCs from HC and RA were stimulated with 100 ng/mL LPS in the presence or absence of 100 ng/mL lTSLP for 1h. Western blots to examine the expression level of p-STAT5, normalized to β-actin. Relative p-STAT5 expression was quantified in **(G)**. **(H)** PBMCs from RA patients were treated with 50 μM STAT5 inhibitor 1h before being stimulated with LPS (100 ng/mL) and lTSLP (100 ng/mL) for RT-PCR detection of *Il-1β*, *Il-6*, *Il-8*, and *Il-10* mRNA expression. P values were measured by unpaired two-tailed Student’s t-test. *P < 0.05, **P < 0.01, ***P < 0.001, ****P < 0.0001. ns, no significant. Error bar represent standard deviation.

Seeing as lTSLP is the primary isoform that exhibits inflammatory cytokine features in a variety of diseases, we postulated that it might possess a pro-inflammatory effect in RA. We cultured PBMCs from different cohorts in the presence or absence of lTSLP under LPS treatment to discern the pro- and anti-inflammatory cytokine expression and revealed that the mRNA expression of cytokines IL-1β, IL-6, IL-8, and IL-10 was upregulated in response to LPS in PBMCs of RA patients and healthy subjects. Astonishingly, lTSLP alone can induce proinflammatory cytokines IL-1β (**Figure 4A**), IL-6 (**Figure 4B**), and IL-8 (**Figure 4C**) mRNA expression in PBMCs from HC or RA patients, while substantially suppressing the anti-inflammatory cytokine IL-10 mRNA expression (**Figure 4D**). Furthermore, lTSLP also enhanced the LPS-induced proinflammatory cytokines IL-1β (**Figure 4A**), IL-6 (**Figure 4B**), and IL-8 (**Figure 4C**) mRNA expression, but inhibited IL-10 mRNA expression upon LPS stimulation (**Figure 4D**). Blocking lTSLP production with a neutralizing antibody decreased proinflammatory cytokine mRNA expression of IL-1β, IL-6, and IL-8 but increased IL-10 mRNA expression (**Figure 4E**). As the molecular pathway of lTSLP signaling involves binding to its receptor complex TSLPR and the IL-7Rα, which activates the downstream signal transducer and activator of transcription 5 (STAT5) (14), we thus investigated the STAT5 event in lTSLP-treated PBMCs of HC and RA patients in the presence or absence of LPS stimulation. As expected, lTSLP alone can trigger STAT5 activation and strongly enhance LPS-mediated STAT5 phosphorylation in PBMCs of HC and RA patients (**Figures 4F, G**). However, the beneficial role of lTSLP in enhancing LPS-induced RA inflammatory responses was markedly abolished after STAT5 inhibitor treatment (**Figure 4H**). Therefore, these results support a pro-inflammatory role of lTSLP in the pathogenesis of RA in a STAT5 signaling-dependent way.

## Discussion

In this study, we presented *in vitro* evidence of the potential associations of TSLP isoforms lTSLP and sTSLP in RA and revealed the molecular mechanisms underlying which lTSLP contributes to detrimental effects in RA by promoting inflammatory responses. Both lTSLP and sTSLP expression are increased in RA, with a distinct profile in which lTSLP is preferentially expressed in active RA patients and sTSLP is more likely to be expressed in inactive RA cases and healthy cohorts. Furthermore, mRNA levels of lTSLP in PBMCs of RA were determined to be linked to disease activity and RA inflammation. The enhancement of inflammation events, which is a pathological hallmark of RA pathogenesis, was amplified by lTSLP in a STAT5 activation-dependent manner.

Although cumulative evidence has implicated the broad pathophysiologic profile of TSLP in inflammatory and autoimmune diseases such as RA (18–21), this is the first study to investigate whether the existence of two isoforms of TSLP, referred to as lTSLP and sTSLP, is distinctly expressed in the pathophysiology of RA and whether their expression is correlated with disease activity in RA. We revealed that the lTSLP and sTSLP mRNA levels were substantially higher in RA patients compared with healthy controls, despite the fact that lTSLP is the predominant variant in RA and sTSLP is preferentially expressed in healthy controls. Moreover, increased lTSLP expression was correlated with DAS28 and ESR, which are reliable biochemical indicators of the acute phase reaction in RA, yet not with other laboratory parameters including RF, CRP, C3, C4, IgG, IgA, IgM, Anti-CCP, Anu A, Anti-dsDNA, and G6PI. Consistent with these observations, lTSLP expression on PBMCs was increased in tandem with the DAS28 increase, whilst sTSLP expression on PBMCs was increased inversely with the DAS28 increase. Moreover, effective RA treatment can restrain lTSLP expression in PBMCs from RA patients, especially active RA patients. Therefore, these findings demonstrate that lTSLP could be employed as a potential biomarker to monitor disease activity in RA. Indeed, these findings are in agreement with previously published research that sTSLP is constitutively expressed by the tissues of the healthy oral cavity, skin, and gut and is downregulated with inflammation, whereas lTSLP is absent in healthy tissues but is upregulated in individuals with atopic dermatitis, Crohn’s disease, and asthma (20, 23–25, 28). lTSLP upregulation can be attributed to a variety of environmental factors and inflammatory conditions, such as viruses, microbes, helminths, allergen sources, cigarette smoke, chemicals, and inflammatory cytokines (9, 11, 20), and it would be fascinating to further study the source of lTSLP expression in RA.

RA is characterized by synovitis and systemic inflammation in which a complex network of multiple cytokines (TNF-a, IL-1β, IL-6, IL-8, etc.) is known to be involved in tissue damage (4, 29). Consequently, biologics that target IL-1β or IL-6 or IL-8 for the treatment of RA have been extensively studied and have profoundly changed RA treatment strategy (29–31). In line with the evidence, our results revealed the proinflammatory cytokines IL-1β, IL-6, and IL-8 mRNA levels in PBMCs of RA are highly increased compared with healthy controls, indicating they are essential for the systemic inflammation in RA. Interestingly, lTSLP expression was associated with IL-1β, IL-6, and IL-8 but negatively with IL-10. It has been widely reported that the elevation of proinflammatory cytokines (IL-1β, IL-6, and IL-8) could promote immune cells to migrate to inflammation sites and take part in the progression of RA inflammation (4, 31), indicating the potentially harmful effects of lTSLP in aggravating the disease situation of patients with RA. To verify this hypothesis, we investigated the function of lTSLP in RA PBMCs *in vitro* and found an individual proinflammatory role for lTSLP and a synergic effect of lTSLP and LPS on promoting the expression of pro-inflammatory cytokines (IL-1β, IL-6, and IL-8). However, lTSLP inhibits IL-10 expression, implying that lTSLP fails on induce anti-inflammatory activity. Several studies have demonstrated the ability of lTSLP to regulate pro-inflammatory cytokine production and promote inflammatory disorders, presumably through STAT5 activation. Here, we confirmed that lTSLP can induce STAT5 phosphorylation in PBMCs from RA and enhance LPS-induced STAT5 activation. These findings highlighted the model that environmental conditions upregulate lTSLP expression in RA and, subsequently, lTSLP contributes to RA systemic inflammatory responses *via* STAT5 phosphorylation.

Over all, we demonstrated two distinct associations with RA for lTSLP and sTSLP isoforms, lTSLP predominantly expressed in active patients with RA and is associated with disease activity as well as inflammatory cytokines, whereas sTSLP is mainly expressed in inactive patients with RA and healthy subjects. Moreover, our finding suggests that lTSLP is a potent inducer of RA inflammation, which involves disruption of cytokine balance towards proinflammatory cytokine responses *via* a STAT5-dependent signaling pathway. Finally, our data emphasize that lTSLP could serve as a biomarker for the diagnosis of RA and it might become a therapeutic intervention target for RA, but its other roles in the pathogenesis of RA still need to be clarified.

## Materials and methods

### Subjects and blood samples

A total of 68 RA patients and 66 healthy individuals were enrolled at Peking University Shenzhen Hospital. The study was approved by the Review Board for Shenzhen University Medical School, and informed consent was obtained from all subjects. Patients diagnosed with RA fulfill the 1987 and 2010 criteria of the American College of Rheumatology (32, 33). DAS28 was used to assess RA disease activities, with DAS28 > 2.8 indicating acute RA courses and DAS28 ≤ 2.8 indicating inactive RA courses (34). At the time of sample collection, 38 patients were receiving prednisone and 30 were not. **Table 1** shows the demographic and clinical information of RA patients and healthy controls.

Blood samples were obtained from all participants during the study. PBMCs from whole blood were isolated by using the Human Lymphocyte Separation Medium (Dakewei, 7111011) after density gradient centrifugation, according to the manufacturer’s instructions. All samples were processed in under 3 hours. PBMCs were cultured or stored at -80 °C until RNA extraction.

### Cell culture condition

PBMCs were grown in RPMI 1640 (Gibco, C11995500BT) medium supplemented with 10% inactivated fetal bovine serum (FBS) (Gibco, 10270106) and 1% penicillin/streptomycin (PS) (Gibco, 15140-122), maintaining at 37 °C with 5% CO_2_. These cells were cultured in 12-well plates at a density of 2 × 10^5^ cells/ml. Following that, these cells were treated under identical conditions: For RNA extraction and cytokine detection, PBMCs of RA and healthy controls were stimulated with or without LPS (100 ng/ml) and co-stimulated with lTSLP (100 ng/ml) for 24h. For protein extraction and Western blot analysis, PBMCs from HC were stimulated with 100 ng/mL LPS in the presence or absence of 100 ng/mL lTSLP for 1h.

### *In vitro* neutralization and blocking experiments

For lTSLP neutralization, PBMCs from RA patients were cultured with 0.25 µg/ml of anti-human TSLP neutralizing antibody (R&D, AF1398) or IgG antibody control (R&D, 5-001-A) for 24h, and cells were collected until RNA extraction. For STAT5 inhibition, PBMCs from RA patients were treated with or without 50 μM of STAT5 inhibitor (Santa Cruz, sc-355979) for 1h before LPS and lTSLP stimulation for 24h. Cells were analyzed by RT-qPCR for cytokine expression.

### Quantitative real-time polymerase chain reaction

The total RNA of PBMCs was extracted using a Trizol reagent (Takara, 9109) as described previously (35). cDNA synthesis was reverse transcribed using the Revert Aid First Strand cDNA Synthesis Kit (Thermo Fisher, K1622). After cDNA obtaining, qPCR analysis was detected using Tip Green qPCR SuperMix (Transgen, AQ141-01) on a Real-Time PCR System (Bio-Rad, CFX96). Results were calculated using the (2^-ΔCt^) or log (2^-ΔCt^) method relative to the expression of *β-actin*. The primer sequences that were used in subsequent experiments are summarized below. *β-actin* forward (fwd) 5’-TCCTCTCCCAAGTCCACACAGG-3’, *β-actin* reverse (rev) 5’-GGGCACGAAGGCTCATCATTC-3’; *lTSLP* fwd 5’-CACCGTCTCTTGTAGCAATCG-3’, *lTSLP* rev 5’-TAGCCTGGGCACCAGATAGC-3’; *sTSLP* fwd 5’-CGTAAACTTTGCCGCCTATGA-3’, *sTSLP* rev 5’-TTCTTCATTGCCTGAGTAGCATTTAT-3’; *Il-1β* fwd 5’-CCACAGACCTTCCAGGAGAAT-3’, *Il-1β* rev 5’-GTGCACATAAGCCTCGTTATCC-3’; *Il-6* fwd 5’-AACCTGAACCTTCCAAAGATGG-3’, *Il-6* rev 5’-TCTGGCTTGTTCCTCACTACT-3’; *Il-8* fwd 5’-GAGAGTGATTGAGAGTGGACCAC-3’, *Il-8* rev 5’-CACAACCCTCTGCACCCAGTTT-3’; *Il-10* fwd 5’-TCTCCGAGATGCCTTCAGCAGA-3’, *Il-10* rev 5’-TCAGACAAGGCTTGGCA ACCCA-3’.

### Western blotting

PBMCs were washed with PBS, and lysed with RIPA buffer (Solarbio, R0010), and quantified using a BCA assay kit (Beyotime, P0012) according to the manufacturer’s instructions. The samples were then boiled 5 minutes at 95 °C in a 5 × protein loading buffer (Meilunbio, MA0003-D). An equivalent quantity of protein samples was loaded onto a 10% sodium dodecyl sulfate-polyacrylamide gel electrophoresis gel (Beyotime, P0012AC) and subsequently transferred to a polyvinylidene fluoride (PVDF) membrane (Merck Millipore, IPVH15150) using WB transfer buffer (Solarbio, D1060). After blocking non-specific binding with 5% skimmed milk (BioFroxx, 1172GR500), rabbit anti-STAT5 (phospho Y694) monoclonal antibody (Abcam, ab32364) and mouse β-actin primary antibody (Abcam, ab8226) were incubated overnight at 4°C. After washing with Tris-buffered solution (Solarbio, T1080) for three times, the blots were incubated with species-specific secondary antibodies (Abcam, ab6721/ab6728) for two hours at room temperature. After five additional washes with Tris-buffered solution containing 0.01% Tween 20, the protein bands were visualized using an ECL reagent kit (Solarbio, PE0010) based on the manufacturer’s instructions (Carestream, USA). Images were captured with a Chemiluminescence Imaging System (CLINX, ChemiScope 6100 Touch, China).

### Statistical analysis

All data were analyzed by GraphPad Prism V.8.0.2 software (GraphPad Software, San Diego CA, USA). Statistical significance was evaluated using unpaired two-tailed Student’s t-test or two-way ANOVA. Correlation analysis was measured by Spearman’s non-parametric test. The results are presented as means ± SD. Asterisks denote a significant difference: *P < 0.05; **P < 0.01; ***P < 0.001; ****P < 0.0001; ns, no significant.

## Data availability statement

The original contributions presented in the study are included in the article/supplementary material. Further inquiries can be directed to the corresponding authors.

## Ethics statement

The studies involving human participants were reviewed and approved by Review Board for Shenzhen University Medical School. The patients/participants provided their written informed consent to participate in this study. Written informed consent was obtained from the individual(s) for the publication of any potentially identifiable images or data included in this article.

## Author contributions

WL, CL, and JD carried out the experiments, analyzed the data, and prepared the figure. JH, LW, and XS contributed to data collection and data analysis. ZH, and XX contributed to coordinating the study and revising the draft manuscript. LY designed the experiments, wrote and revised the paper, and supervised the project. All authors contributed to the article and approved the submitted version.

## References

[1] Firestein GS. Evolving concepts of rheumatoid arthritis. Nature (2003) 423(6937):356–61. doi: 10.1038/nature01661 12748655

[2] Smolen JS Aletaha D McInnes IB. Rheumatoid arthritis. Lancet (2016) 388(10055):2023–38. doi: 10.1016/S0140-6736(16)30173-8 27156434

[3] Scott DL Wolfe F Huizinga TW. Rheumatoid arthritis. Lancet (2010) 376(9746):1094–108. doi: 10.1016/S0140-6736(10)60826-4 20870100

[4] McInnes IB Schett G. Cytokines in the pathogenesis of rheumatoid arthritis. Nat Rev Immunol (2007) 7(6):429–42. doi: 10.1038/nri2094 17525752

[5] Noack M Miossec P. Selected cytokine pathways in rheumatoid arthritis. Semin Immunopathol (2017) 39(4):365–83. doi: 10.1007/s00281-017-0619-z 28213794

[6] Kashyap M Rochman Y Spolski R Samsel L Leonard WJ. Thymic stromal lymphopoietin is produced by dendritic cells. J Immunol (2011) 187(3):1207–11. doi: 10.4049/jimmunol.1100355 PMC3140600 21690322

[7] Biancheri P Di Sabatino A Rescigno M Giuffrida P Fornasa G Tsilingiri K. Abnormal thymic stromal lymphopoietin expression in the duodenal mucosa of patients with coeliac disease. Gut (2016) 65(10):1670–80. doi: 10.1136/gutjnl-2014-308876 PMC5036244 26342013

[8] Calven J Yudina Y Hallgren O Westergren-Thorsson G Davies DE Brandelius A. Viral stimuli trigger exaggerated thymic stromal lymphopoietin expression by chronic obstructive pulmonary disease epithelium: Role of endosomal TLR3 and cytosolic RIG-i-like helicases. J Innate Immun (2012) 4(1):86–99. doi: 10.1159/000329131 21691053

[9] Kato A Favoreto S Jr. Avila PC Schleimer RP. TLR3- and Th2 cytokine-dependent production of thymic stromal lymphopoietin in human airway epithelial cells. J Immunol (2007) 179(2):1080–7. doi: 10.4049/jimmunol.179.2.1080 PMC2220044 17617600

[10] Lee HC Ziegler SF. Inducible expression of the proallergic cytokine thymic stromal lymphopoietin in airway epithelial cells is controlled by NFkappaB. Proc Natl Acad Sci U.S.A. (2007) 104(3):914–9. doi: 10.1073/pnas.0607305104 PMC1783414 17213320

[11] Corren J Ziegler SF. TSLP: from allergy to cancer. Nat Immunol (2019) 20(12):1603–9. doi: 10.1038/s41590-019-0524-9 31745338

[12] Park LS Martin U Garka K Gliniak B Di Santo JP Muller W. Cloning of the murine thymic stromal lymphopoietin (TSLP) receptor: Formation of a functional heteromeric complex requires interleukin 7 receptor. J Exp Med (2000) 192(5):659–70. doi: 10.1084/jem.192.5.659 PMC2193276 10974032

[13] Pandey A Ozaki K Baumann H Levin SD Puel A Farr AG. Cloning of a receptor subunit required for signaling by thymic stromal lymphopoietin. Nat Immunol (2000) 1(1):59–64. doi: 10.1038/76923 10881176

[14] Rochman Y Kashyap M Robinson GW Sakamoto K Gomez-Rodriguez J Wagner KU. Thymic stromal lymphopoietin-mediated STAT5 phosphorylation *via* kinases JAK1 and JAK2 reveals a key difference from IL-7-induced signaling. Proc Natl Acad Sci U.S.A. (2010) 107(45):19455–60. doi: 10.1073/pnas.1008271107 PMC2984176 20974963

[15] Ye L Schnepf D Becker J Ebert K Tanriver Y Bernasconi V. Interferon-lambda enhances adaptive mucosal immunity by boosting release of thymic stromal lymphopoietin. Nat Immunol (2019) 20(5):593–601. doi: 10.1038/s41590-019-0345-x 30886417

[16] Ye L Schnepf D Ohnemus A Ong LC Gad HH Hartmann R. Interferon-lambda improves the efficacy of intranasally or rectally administered influenza subunit vaccines by a thymic stromal lymphopoietin-dependent mechanism. Front Immunol (2021) 12:749325. doi: 10.3389/fimmu.2021.749325 34659250 PMC8511795

[17] Hartgring SA Willis CR Dean CE Jr. Broere F van Eden W Bijlsma JW. Critical proinflammatory role of thymic stromal lymphopoietin and its receptor in experimental autoimmune arthritis. Arthritis Rheum (2011) 63(7):1878–87. doi: 10.1002/art.30336 21391201

[18] Eckhardt J Dobbeler M Konig C Kuczera K Kuhnt C Ostalecki C. Thymic stromal lymphopoietin deficiency attenuates experimental autoimmune encephalomyelitis. Clin Exp Immunol (2015) 181(1):51–64. doi: 10.1111/cei.12621 25753260 PMC4469155

[19] Moret FM Hack CE van der Wurff-Jacobs KM Radstake TR Lafeber FP van Roon JA. Thymic stromal lymphopoietin, a novel proinflammatory mediator in rheumatoid arthritis that potently activates CD1c+ myeloid dendritic cells to attract and stimulate T cells. Arthritis Rheumatol (2014) 66(5):1176–84. doi: 10.1002/art.38338 24782181

[20] Varricchi G Pecoraro A Marone G Criscuolo G Spadaro G Genovese A. Thymic stromal lymphopoietin isoforms, inflammatory disorders, and cancer. Front Immunol (2018) 9:1595. doi: 10.3389/fimmu.2018.01595 30057581 PMC6053489

[21] Markovic I Savvides SN. Modulation of signaling mediated by TSLP and IL-7 in inflammation, autoimmune diseases, and cancer. Front Immunol (2020) 11:1557. doi: 10.3389/fimmu.2020.01557 32849527 PMC7396566

[22] Harada M Hirota T Jodo AI Doi S Kameda M Fujita K. Functional analysis of the thymic stromal lymphopoietin variants in human bronchial epithelial cells. Am J Respir Cell Mol Biol (2009) 40(3):368–74. doi: 10.1165/rcmb.2008-0041OC 18787178

[23] Tsilingiri K Fornasa G Rescigno M. Thymic stromal lymphopoietin: To cut a long story short. Cell Mol Gastroenterol Hepatol (2017) 3(2):174–82. doi: 10.1016/j.jcmgh.2017.01.005 PMC5331833 28275684

[24] Fornasa G Tsilingiri K Caprioli F Botti F Mapelli M Meller S. Dichotomy of short and long thymic stromal lymphopoietin isoforms in inflammatory disorders of the bowel and skin. J Allergy Clin Immunol (2015) 136(2):413–22. doi: 10.1016/j.jaci.2015.04.011 PMC4534776 26014813

[25] Dong H Hu Y Liu L Zou M Huang C Luo L. Distinct roles of short and long thymic stromal lymphopoietin isoforms in house dust mite-induced asthmatic airway epithelial barrier disruption. Sci Rep (2016) 6:39559. doi: 10.1038/srep39559 27996052 PMC5171874

[26] Bjerkan L Schreurs O Engen SA Jahnsen FL Baekkevold ES Blix IJ. The short form of TSLP is constitutively translated in human keratinocytes and has characteristics of an antimicrobial peptide. Mucosal Immunol (2015) 8(1):49–56. doi: 10.1038/mi.2014.41 24850429

[27] Yu C Huang W Zhou Z Liang S Zhou Z Liu J. Short isoform thymic stromal lymphopoietin reduces inflammation and aerobic glycolysis of asthmatic airway epithelium by antagonizing long isoform thymic stromal lymphopoietin. Respir Res (2022) 23(1):75. doi: 10.1186/s12931-022-01979-x 35351157 PMC8966346

[28] Martin Mena A Langlois A Speca S Schneider L Desreumaux P Dubuquoy L. The expression of the short isoform of thymic stromal lymphopoietin in the colon is regulated by the nuclear receptor peroxisome proliferator activated receptor-gamma and is impaired during ulcerative colitis. Front Immunol (2017) 8:1052. doi: 10.3389/fimmu.2017.01052 28928735 PMC5591373

[29] Feldmann M Brennan FM Maini RN. Role of cytokines in rheumatoid arthritis. Annu Rev Immunol (1996) 14:397–440. doi: 10.1146/annurev.immunol.14.1.397 8717520

[30] Huang J Fu X Chen X Li Z Huang Y Liang C. Promising therapeutic targets for treatment of rheumatoid arthritis. Front Immunol (2021) 12:686155. doi: 10.3389/fimmu.2021.686155 34305919 PMC8299711

[31] McInnes IB Buckley CD Isaacs JD. Cytokines in rheumatoid arthritis - shaping the immunological landscape. Nat Rev Rheumatol (2016) 12(1):63–8. doi: 10.1038/nrrheum.2015.171 26656659

[32] Arnett FC Edworthy SM Bloch DA McShane DJ Fries JF Cooper NS. The American rheumatism association 1987 revised criteria for the classification of rheumatoid arthritis. Arthritis Rheum (1988) 31(3):315–24. doi: 10.1002/art.1780310302 3358796

[33] Aletaha D Neogi T Silman AJ Funovits J Felson DT Bingham CO 3rd. 2010 rheumatoid arthritis classification criteria: An American college of Rheumatology/European league against rheumatism collaborative initiative. Arthritis Rheum (2010) 62(9):2569–81. doi: 10.1002/art.27584 20872595

[34] van Tuyl LH Vlad SC Felson DT Wells G Boers M. Defining remission in rheumatoid arthritis: Results of an initial American college of Rheumatology/European league against rheumatism consensus conference. Arthritis Rheum (2009) 61(5):704–10. doi: 10.1002/art.24392 PMC2681785 19405006

[35] Liu Y Song X Li C Hu H Li W Wang L. Chrysin ameliorates influenza virus infection in the upper airways by repressing virus-induced cell cycle arrest and mitochondria-dependent apoptosis. Front Immunol (2022) 13:872958. doi: 10.3389/fimmu.2022.872958 35432374 PMC9009290

